# A randomized controlled trial of an internet-based intervention for alcohol abusers

**DOI:** 10.1111/j.1360-0443.2009.02726.x

**Published:** 2009-12

**Authors:** John A Cunningham, T Cameron Wild, Joanne Cordingley, Trevor van Mierlo, Keith Humphreys

**Affiliations:** 1 Centre for Addiction and Mental HealthToronto, Ontario, Canada; 2 University of TorontoToronto, Ontario, Canada; 3 University of AlbertaEdmonton, Alberta, Canada; 4 Evolution Health Systems Inc.Toronto, Ontario, Canada; 5 Veterans Affairs and Stanford University Medical CentersStanford, CA, USA

**Keywords:** Alcohol, brief intervention, internet, problem drinking, randomized controlled trial

## Abstract

**Objective:**

Misuse of alcohol imposes a major public health cost, yet few problem drinkers are willing to access in-person services for alcohol abuse. The development of brief, easily accessible ways to help problem drinkers who are unwilling or unable to seek traditional treatment services could therefore have significant public health benefit. The objective of this project is to conduct a randomized controlled evaluation of the internet-based Check Your Drinking (CYD) screener (http://www.CheckYourDrinking.net).

**Method:**

Participants (*n* = 185) recruited through a general telephone population survey were assigned randomly to receive access to the CYD, or to a no-intervention control group.

**Results:**

Follow-up rates were excellent (92%). Problem drinkers provided access to the CYD displayed a six to seven drinks reduction in their weekly alcohol consumption (a 30% reduction in typical weekly drinking) at both the 3- and 6-month follow-ups compared to a one drink per week reduction among control group respondents.

**Conclusions:**

The CYD is one of a growing number of internet-based interventions with research evidence supporting its efficacy to reduce alcohol consumption. The internet could increase the range of help-seeking options available because it takes treatment to the problem drinker rather than making the problem drinker come to treatment.

## INTRODUCTION

Problem drinking is one of the three leading contributors to preventable death [1] and increases significantly the likelihood of experiencing morbidity, trauma, casualties and violence [2–4]. The ratio of problem drinkers to those seriously dependent on alcohol is about 4 : 1 [4]. As Cahalan has noted, ‘clinically defined alcoholics constitute only a relatively small proportion of those whose drinking creates significant problems for themselves and society’ (p. 363) [5]. The challenge in attempting to help problem drinkers is that most will never seek traditional alcohol treatment [6,7]. Consequently, efforts are under way to take treatment to problem drinkers if they will not come to treatment. One option is to have primary care physicians administer a brief intervention to problem drinkers [8–10]. This method has met with some success; however, there are concerns about time, competing priorities and patient interest that may preclude the widespread adoption of this intervention option [11,12].

What other means of helping problem drinkers exist? An ideal option would involve delivery of alcohol interventions so that they could be accessed freely, 24 hours a day, in the comfort of the person's home and free of geographic restrictions regarding treatment availability. If such an option were available, problem drinkers could access it on their own initiative or upon their physician's recommendation. New technologies may make such interventions possible. Primarily, the internet is becoming a common feature of everyday life, and within the next decade will probably be used as widely as the telephone or television [13]. Already, many problem drinkers—primarily young people—have access to the internet. In a recent general population survey, 81% of problem drinkers in Ontario, Canada, had home access to the internet [14]. Many problem drinkers also express an interest in receiving help over the internet, making this medium an opportunity to help problem drinkers who will not seek traditional face-to-face treatment [15]. Although internet-based interventions (IBIs) may never be as effective as a face-to-face encounter with a skilled clinician, the reality is that most problem drinkers will never receive a face-to-face intervention, in particular with a skilled clinician [6,7]. The challenge then is to design interventions that can be accessed by problem drinkers and then to demonstrate their efficacy. As problem drinking is prevalent, validated IBIs, made freely available to all those in need, could greatly broaden the base of treatment for alcohol problems and may prove to be a useful tool for primary care.

IBIs for problem drinkers have been in existence for more than a decade [16]. The majority were developed and evaluated in college settings [17–19], but others have been designed for general population use [20–23]. Many other IBIs exist with little or no research evaluation [24]. An earlier version of the IBI used in this trial, the Check Your Drinking screener (CYD) [16], has been subjected to three small randomized controlled trials. One was with young adults in the work-place [25], and two were with college students (intercollegiate athletes [26] and students mandated for drinking infractions [27]). In these trials, the CYD intervention was administered in a face-to-face setting and short-term impact on drinking was measured (1–3 months). In all three trials, problem drinkers who received the CYD intervention displayed significant reductions in alcohol consumption compared to those in the control conditions. Only one randomized controlled trial has been published to date in which the intervention was administered over the internet and whose participants were adult problem drinkers from the general population [22]. In this study, respondents in the intervention condition were provided access to a Dutch language website containing a multi-component cognitive behavioural intervention. Respondents provided access to the intervention website displayed significant reductions in drinking compared to controls at a 6-month follow-up. The present study will add to this literature by assessing the impact of a brief personalized feedback intervention, delivered over the internet, in an adult population.

There are several challenging aspects to consider in evaluating an IBI—the population, the setting and the internal validity of the research trial [28]. The target population of interest is the general population of problem drinkers who are not treatment seekers. Most research trials evaluating the efficacy of IBIs were conducted with college students [24]. These studies have provided support for the effectiveness of IBIs with college students, but the results cannot be assumed to generalize to the entire population of problem drinkers. The challenge of setting refers to the fact that most research trials are conducted in face-to-face settings, but IBIs are designed for use over the internet and in the person's home. Given that many people tend to move quickly from one internet page to another, it is important to design an IBI so that it is engaging and brief. Further, when evaluating the efficacy of an IBI, it cannot be assumed that an IBI that is effective when administered in a face-to-face setting will also work when it is accessed remotely (e.g. in the person's home or other location of his or her choice; will the participant even engage with the IBI if nobody is there with them?) [21]. Finally, the primary concern with internal validity in research trials of IBIs is poor follow-up rates, with many trials following-up less than 40% of participants [29]. Thus, the challenge of this trial was to recruit a sample of non-treatment-seeking problem drinkers from the general population, administer the intervention in a naturalistic setting (i.e. not face-to-face in a laboratory) and then obtain a good follow-up rate to evaluate its efficacy. It was predicted that problem drinkers who were provided access to the CYD would display improved drinking outcomes compared to those in a no-intervention control group at 3- and 6-month follow-ups. Further, based on earlier work with the CYD intervention [25–27], it was predicted that drinking reductions would be observed among problem drinkers, but not with low-risk drinking recipients.

## METHODS

### Summary of design

Recruitment for this study took advantage of an ongoing general population telephone survey that collected socio-demographic and drinking baseline data from a large, randomly selected probability sample of Ontario adults. Drinkers who met minimal criteria for risky alcohol consumption [score of 4 or more on the 3 consumption items from the Alcohol use Disorders Identification Test (AUDIT-C)][30] were identified and were asked: ‘The next question asks about self-help materials for drinkers that the Centre for Addiction and Mental Health may provide in the future. Would you be interested in a confidential program that you could access on the internet, free-of-charge, that would allow you to check your drinking and compare it to other Canadians?’. At the end of the survey, all drinkers who said they were interested in a computerized summary and who reported having home access to the internet were told: ‘Researchers at the Centre for Addiction and Mental Health are currently developing self-help materials for drinkers. They are looking for regular drinkers to participate in a study to help revise and evaluate an internet program that would compare your drinking to other Canadians. The study would involve looking at some materials and then filling out brief surveys in 3, 6, and 12 months’ time. You would be paid $60 for your participation. Would you be interested in receiving a description of this study to see if you would like to participate?'. Interested respondents (*n* = 397) provided their name, address and telephone number. They were then sent a cover letter and consent form explaining the study, along with a supplementary baseline questionnaire. Those respondents agreeing to participate (*n* = 185, 47% of those indicating interest) signed and returned a copy of the consent form along with the baseline questionnaire (i.e. after complete description of the study to the respondents, written informed consent was obtained). Respondents were then randomized into one of two conditions: (i) an internet personalized alcohol feedback condition (intervention condition); or (ii) a no-intervention control condition. Randomization was conducted using a random numbers list (odd numbers for condition one and even numbers for condition two) with no stratification. All respondents were followed-up in 3 and 6 months' time to determine changes in drinking status (respondents were sent a $20 cheque along with each of the follow-up surveys). The conduct of this study was approved by the standing ethics review committee of the Centre for Addiction and Mental Health.

### Experimental groups

#### Intervention condition: internet personalized alcohol feedback

A letter was sent to respondents in the intervention condition thanking them for agreeing to participate in the study and containing the internet address (URL) for the CYD and a password to allow access. Respondents were asked to review the CYD and were informed that they would be asked their impressions of the CYD on the 3-month follow-up. If respondents had not accessed the website within a month of receiving the initial invitation letter, they were sent a reminder letter asking them to log on.

**Figure 1:**
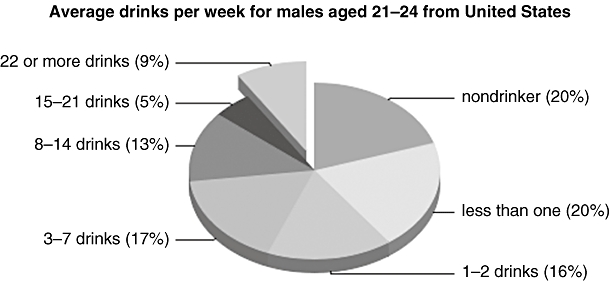
Example feedback from the Check Your Drinking screener

The materials employed for the CYD have been modelled after the Drinker's Check-up [31–33] and the Fostering Self-Change intervention [34]. After completing a brief online assessment, participants receive a ‘Personalized Drinking Profile’. The core elements of the CYD are: (i) normative feedback pie charts that compare the participant's drinking to others of the same age, sex and country of origin (for Canada, the United States and the United Kingdom; more country data to be added; see Fig. 1 for an example of this feedback); and (ii) a summary of the participant's severity of alcohol problems. The contents of the CYD intervention are described in more detail elsewhere [35]. The CYD is brief, taking less than 10 minutes to complete. In response to feedback from the original version of the CYD, this trial employed a modified version so that participants who reported drinking less than once per week were not asked about their drinking during a typical week and did not receive feedback charts discussing drinking during a typical week (version 2.0 of the Check Your Drinking program). To gain a clearer picture of the IBI, the reader is invited to access the public copy of the program (http://www.CheckYourDrinking.net).

#### No intervention control group

Respondents in the control group did not receive any feedback materials. Instead they were sent a list of the informational components that could be included in a computerized summary for drinkers (this request made sense, as respondents were informed that the purpose of the study was to help ‘revise and evaluate self-help materials’). A letter accompanied this list thanking them for agreeing to participate in the study, asking them to consider how useful they might find the different components that could be included in a computerized summary for drinkers, and telling them that they would be asked for their opinions on the 3-month follow-up. The listed components were the same as those included in the CYD intervention (e.g. a chart that compares the user's drinking to other Canadians of the same age and sex).

### Baseline assessment

Demographic characteristics including age, sex, marital status, education, gross family income and employment status were collected on the initial random digit dialling telephone survey (this survey also contained the three AUDIT-C items to identify risky drinkers). All other items were collected on the paper survey mailed out with the consent form. These items included the AUDIT [36,37]. Respondents' drinking was also assessed using the period-specific normal week approach [38]. This method asks respondents for their alcohol consumption during a typical week (i.e. usual number of drinks on each day of a typical week).

#### Data analysis

Before conducting the outcome analyses, drinking data at baseline, 3-month and 6-month follow-up were examined for their distributional characteristics. Missing data were replaced with the corresponding baseline data (note that, of 185 respondents, 10 had missing data at 3 months and 12 had missing data at 6 months; analyses were also conducted without replacement of missing data with similar results to those reported here). Drinking variables were also trimmed by replacing any outliers beyond three standard deviations with the next highest value (this resulted in drinking variables that approached normal distributional characteristics). Two composite outcome measures were chosen that incorporated most of the essential components as outcome measures for a brief intervention. The primary outcome measure was number of drinks in a typical week (sum of number of drinks reported on each day of a typical week) [38,39]. The second measure was the AUDIT-C (a composite measure of the first three items on the AUDIT—typical frequency of consumption, drinks per drinking day and frequency of five or more drinks per occasion). The AUDIT-C has a possible score range of 0–12, with higher scores reflecting more severe drinking problems [30].

Analyses were conducted using 2 × 2 × 3 repeated-measures analyses of variance (ANOVAs). The within-subjects variable was time of follow-up (baseline, 3-month and 6-month follow-up). Intervention condition (received internet address or control group) and baseline problem drinking status (problem drinkers: score on the full AUDIT of 11 or more versus low-risk drinkers: AUDIT score of 4–10) were the between-subjects variables. Baseline problem drinking status was included in the analyses because previous research employing the CYD has found that this intervention only had an impact with problem drinkers [25–27]. A score of 11 or more is one of the suggested cut-off scores for the AUDIT to indicate problem drinking [36]. We adopted the cut-off of 11 on the AUDIT (rather than the lower one of 8 or more) because this produced a median weekly drinking pattern in our respondents that exceeded the recommended level of drinking recommended by the National Institute on Alcohol Abuse and Alcoholism (NIAAA) as the point at which a brief intervention should be administered [40]. It should also be noted that, in addition to the analyses presented here, we also conducted the same analyses with sex included as one of the factors. These analyses were conducted because of concern that our cut-off points in recruitment and our definition of problem drinking did not use differential criteria for males versus females. However, as there were no significant (*P* > 0.05) effects of sex of the respondent in these analyses, they are not presented here. Finally, we chose a conservative intent-to-treat analysis in which respondents in the intervention condition were included in the analysis even if they never accessed the CYD intervention (35 of 92 possible participants did not access the website).

#### Power analysis and changes from original recruitment plans

**Figure 2:**
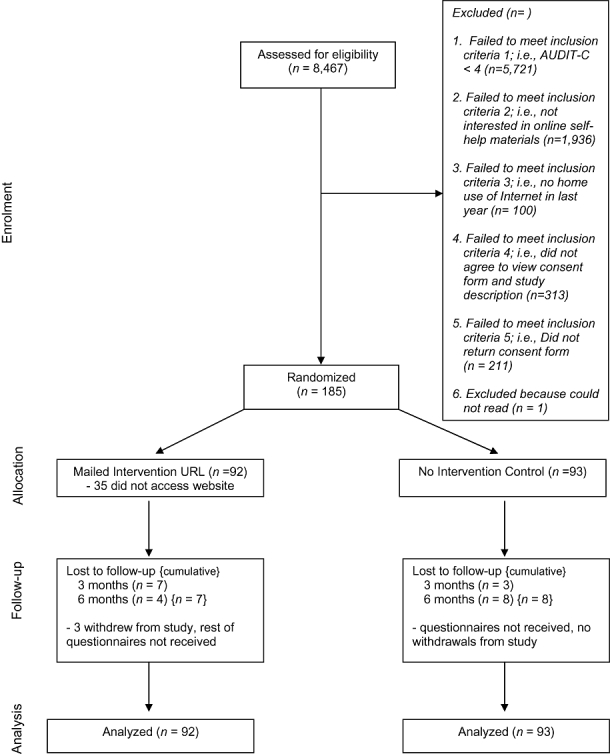
Overview of the internet-based intervention for problem drinkers trial. AUDIT-C: Alcohol Use Disorders Identification Test

The original plans for this study called for recruiting respondents who had AUDIT scores of 8 or more. A power analysis was conducted based on previous research with similar paper-and-pencil versions of this intervention [41] and a total sample size of 170 respondents after attrition was estimated to have a power of 80% to test the hypothesis at the *P* < 0.05 level of significance. However, we were able to include only the three AUDIT-C items in the telephone recruitment survey, as the telephone survey was being conducted for another purpose. Therefore, the senior author of this paper decided to try to recruit as many potential participants as possible using a minimal indicator of risky drinking, i.e. an AUDIT-C score of 4 or more. This decision appears merited in retrospect, as many potential participants who voiced an interest in the study never returned their consent form (this was not anticipated in the initial recruitment plan; see Fig. 2 for recruitment numbers). Nevertheless, sufficient numbers were recruited for the study and having a wider range of severity of drinking problems at baseline allowed us to adopt problem severity as a potential moderator of change based on the research that had emerged since the time of grant submission [25,26].

## RESULTS

Figure 2 provides a chart outlining the recruitment and retention of the 185 respondents in this study using the Consolidated Standards of Reporting Trials (CONSORT) guidelines for reporting randomized trials. Participant retention was excellent, with a 95% follow-up rate at 3 months and a 93% follow-up rate at 6 months. A total of 170 respondents (92%) had complete data at baseline, 3-month and 6-month follow-ups.

For the 2746 respondents who met minimal criteria for risky drinking (i.e. an AUDIT-C score of 4 or more), attrition analyses were conducted to compare the characteristics of potential respondents who screened out at each phase of the telephone recruitment survey (see Table 1). In general, while there were significant differences between groups on most variables due to the large sample sizes involved, the groups did not display large differences in demographic characteristics or on their AUDIT-C scores. The most striking differences were that those who agreed to take part in the study were somewhat younger and more educated than those in the other groups, *F*_(4, 2689)_ = 33.6, *P* < 0.001 and χ^2^ = 47.9, 4 df, *P* < 0.001). Finally, for the 185 respondents enrolled in the trial, attrition analyses were conducted comparing those with complete follow-up data (*n* = 170) to those with at least partial missing follow-up data (*n* = 15). There were no significant differences (*P* > 0.05) in demographic or drinking characteristics between these groups (analyses not shown here).

**Table 1:** Attrition analysis for potential respondents screened out prior to consent (*n* = 2746).

*Variable*	*Not interested in online program (n* = *1936)*	*No home access to internet (n* = *100)*	*Did not agree to view consent form (n* = *313)*	*Did not return consent form (n* = *212)*	*Consented to participate in study (n* = *185)*	*P*
Mean (SD) age	46.8 (16.2)	44.9 (14.8)	39.2 (13.8)	37.6 (12.5)	40.1 (13.4)	0.001
% Male	62.1	65.0	64.5	57.5	53.0	NS
% Some post-secondary education	62.4	51.0	72.0	76.9	77.8	0.001
% Married/common law	60.2	49.0	58.7	53.6	51.4	0.05
% Full/part-time employed	64.4	71.0	69.3	71.2	62.5	NS
% Family income						
<$30 000	10.1	24.0	8.9	7.5	10.3	
$30 000 or more	71.3	64.0	75.7	85.8	83.2	
Don't know/refused	18.6	12.0	15.3	6.6	6.5	0.001
AUDIT-C score^a^	5.5 (1.7)	5.8 (1.7)	5.3 (1.5)	5.9 (1.8)	5.6 (1.7)	0.001

NS: not significant, *P* > 0.05. Each column contains just those potential participants who screened out at each question on telephone recruitment survey (e.g. the 100 potential participants who had no home access to the internet had already indicated that they were interested in an online program, etc).

a Alcohol Use Disorders Identification Test (AUDIT-C) is a composite measure that consists of respondents scores on frequency of drinking, drinks per drinking day and frequency of five or more drinks on one occasion. Scores range from 0 to 12. SD: standard deviation.

Bivariate comparisons were conducted to compare demographic and drinking characteristics between the experimental and the control conditions at baseline (note that bivariate comparisons between experimental and control conditions were also conducted for problem drinker groups and low-risk drinker groups separately). None of these comparisons were significantly different (*P* > 0.05; see Table 2 for a summary of demographic characteristics).

**Table 2:** Demographic characteristics.

*Variable*	*Invervention (n* = *92)*	*Control (n* = *93)*	*Total sample (n* = *185)*
Mean (SD) age	39.5 (13.5)	40.8 (13.4)	40.1 (13.4)
% Male	57.6	48.4	53.0
% Some post-secondary education	78.3	77.4	77.8
% Married/common law	54.3	48.4	51.4
% Full/part-time employed	62.6	62.4	62.5
% Family income			
<$30 000	6.5	14.0	10.3
$30 000–$49 000	16.3	12.9	14.6
$50 000–$79 000	18.5	21.5	20.0
$80 000 or more	48.9	48.4	48.6
Don't know/refused	9.8	3.2	6.5

All bivariate comparisons between groups were not significantly different (*P* > 0.05). SD: standard deviation.

### Outcome analysis

Means for typical weekly drinking and AUDIT-C scores at baseline, 3 and 6 months by intervention condition and problem drinking status are displayed in Table 3. A 2 × 2 × 3 repeated-measures ANOVA compared typical weekly drinking at baseline, 3-month and 6-month follow-ups between intervention condition (received URL for CYD website versus control condition) and problem drinking status at baseline. There was a main effect of time (*F*_(2,180)_ = 13.8, *P* < 0.001, partial eta squared = 0.13), which was qualified by a significant time × intervention interaction (*F*_(2,180)_ = 7.5, *P* = 0.001, partial eta squared = 0.077), and a significant time × baseline problem drinking status interaction (*F*_(2,180)_ = 9.7, *P* < 0.001, partial eta squared = 0.098). These two-way interactions were qualified further by a significant time × intervention × baseline problem drinking status interaction (*F*_(2,180)_ = 7.9, *P* = 0.001, partial eta squared = 0.081). *Post-hoc* analyses revealed that, for problem drinkers, there was a significant reduction in typical weekly drinking (*P* < 0.05) from baseline to 3-month follow-up (average of seven drinks per week reduction) and from baseline to 6-month follow-up (average of six drinks per week reduction) for respondents who were in the intervention condition, but no significant reduction (*P* > 0.05) in drinking from baseline to either time-point for respondents in the control condition (average of one drink per week reduction). Low-risk drinkers did not reveal a significant reduction (*P* > 0.05) in their drinking in either the intervention or the control conditions.

**Table 3:** Mean [standard deviation (SD)] drinking variables at baseline, 3- and 6-month follow-up by study condition (Check Your Drinking Intervention versus control) and problem drinking status (*n* = 185).

	*Problem drinkers^a^*		*Low-risk drinkers^b^*		*Full sample*	
*Time*	*Intervention (n* = *35)*	*Control (n* = *37)*	*Intervention (n* = *57)*	*Control (n* = *56)*	*Intervention (n* = *92)*	*Control (n* = *93)*
Typical weekly drinking [mean (SD) drinks per week]						
Baseline	22.5 (12.6)	19.1 (12.0)	8.7 (4.8)	7.2 (4.0)	13.9 (10.9)	11.9 (10.1)
3-month	15.1 (11.2)	18.4 (12.3)	8.4 (6.4)	6.7 (4.8)	11.0 (9.1)	11.4 (10.3)
6-month	16.0 (11.9)	17.9 (12.5)	8.1 (4.2)	7.3 (5.4)	11.1 (8.9)	11.5 (10.3)
AUDIT-C score^c^[mean (SD)]						
Baseline	8.9 (1.9)	8.1 (2.2)	5.8 (1.2)	5.3 (1.2)	7.0 (2.1)	6.4 (2.1)
3-month	7.5 (2.3)	8.1 (2.3)	5.7 (1.6)	5.1 (1.5)	6.3 (2.1)	6.3 (2.4)
6-month	7.3 (2.6)	7.9 (2.3)	5.6 (1.6)	5.2 (1.6)	6.2 (2.2)	6.3 (2.3)

a Problem drinkers defined as those having an Alcohol Use Disorders Identification Test (AUDIT) score of 11 or more at baseline.

b Low-risk drinkers defined as those having an AUDIT score of 4–10 at baseline.

c AUDIT-C is a composite measure that consists of respondents' scores on frequency of drinking, drinks per drinking day and frequency of five or more drinks on one occasion. Scores range from 0 to 12.

A separate 2 × 2 × 3 repeated-measures ANOVA compared AUDIT-C scores at baseline, 3-month and 6-month follow-ups between intervention condition (received URL for CYD website versus control condition) and problem drinking status at baseline. There was a main effect of time (*F*_(2,180)_ = 8.7, *P* < 0.001, partial eta squared = 0.088) and significant interaction effects for time × intervention (*F*_(2,180)_ = 5.8, *P* = 0.004, partial eta squared = 0.06), time × problem drinking status (*F*_(2,180)_ = 4.5, *P* = 0.013, partial eta squared = 0.047) and time × intervention × problem drinking status (*F*_(2,180)_ = 5.8, *P* = 0.004, partial eta squared = 0.060). As with typical weekly drinking, *post-hoc* analyses revealed that, for problem drinkers, there was a significant reduction in AUDIT-C scores (*P* < 0.05) from baseline to 3-month follow-up and from baseline to 6-month follow-up for respondents who were in the intervention condition, but no significant reduction (*P* > 0.05) in AUDIT-C scores from baseline to either time-point for respondents in the control condition. Low-risk drinkers did not reveal a significant reduction (*P* > 0.05) in their AUDIT-C scores in either the intervention or the control conditions.

## DISCUSSION

The primary goal of this project was to evaluate the efficacy of the Check Your Drinking screener, an internet-based self-help intervention for non-treatment-seeking problem drinkers in the general population. One strength of the study was that it merged population-based methods with a randomized controlled trial. Thus, a general population survey was employed using a random digit dialling method in order to recruit a good cross-section of problem drinkers from the Ontario population (although almost certainly not a representative one). Respondents were then assigned randomly to condition, allowing for causal inference about any differences observed. Results from the trial are promising, with problem drinkers who were provided access to the CYD displaying a six to seven drinks per week reduction in drinking compared to controls (a 30% reduction in typical weekly drinking). The size of this reduction is of the same magnitude as has been observed in face-to-face brief interventions for problem drinking in primary health care settings [42].

The primary limitation of this study is that about one-third of those in the intervention condition never accessed the CYD. Thus, with its conservative intent-to-treat analysis (i.e. respondents assigned to the intervention were included in the analyses whether or not they actually used the intervention), this trial is a comparison between those provided access to the CYD versus those who were not. The fact that many people did not access the CYD intervention (despite having said they would be interested in such a service on the baseline telephone survey) emphasizes the point that providing access to IBIs, or any other intervention, does not guarantee that problem drinkers will use them. Indeed, no one intervention is an ideal solution and a sensible option for promoting access to care is to increase the variety of research-based interventions available so that problem drinkers can choose one (or several) that is suitable to them.

Other limitations of this study include the large number of potential participants who could have agreed to take part in the research but chose not to. This loss of potential participants could, in part, reflect that the current study did not recruit problem drinkers seeking help but rather proactively recruited from the general population [43]. In addition, the current report is limited by a lack of content exploring mechanisms of change (i.e. why does the intervention work?). There is a fairly extensive literature on this topic with college students [26,44], but little or no research exploring these same mechanisms in general population samples.

Nevertheless, we are encouraged by the results of this trial given the methodological rigor of this research. Mainly, the study demonstrated the possibility of attaining an excellent follow-up rate, something that is rare in evaluations of internet-based interventions in a general population context [29]. We speculate that this high follow-up rate reflects at least two elements (beyond the incentive provided to respondents): (i) respondents were not recruited (or followed-up) directly over the internet where the perceived social obligation of following up on a commitment (e.g. to complete a follow-up survey) may not be as obvious as in a telephone conversation (or after signing a paper consent form); and (ii) by the time respondents had agreed to take part in the study, they were probably self-selected to be compliant as they had already gone through so many steps before actually taking part in the trial. Another strength was that the design of the trial allowed the administration of the intervention in a real-life setting (i.e. in their own home or other location of their choice). This procedure increases confidence regarding the generalizability of the results [28]. As problem drinking is common, research evaluated internet-based interventions made freely available to all those in need could greatly broaden the base of treatment for alcohol problems and may prove to be useful tools for primary care.

## Clinical trial registration

ClinicalTrials.gov registration #NCT00367575.
